# Is the Goligher classification a valid tool in clinical practice and research for hemorrhoidal disease?

**DOI:** 10.1007/s10151-022-02591-3

**Published:** 2022-02-09

**Authors:** L. Dekker, I. J. M. Han-Geurts, U. Grossi, G. Gallo, R. Veldkamp

**Affiliations:** 1 Department of Surgery, Amsterdam University Medical Centres, Location AMC, Meibergdreef 9, 1105 AZ Amsterdam, The Netherlands; 2 Department of Surgery, Proctos Kliniek, Bilthoven, The Netherlands; 3 II Surgery Unit, Regional Hospital Treviso, Department of Surgery, Oncology and Gastroenterology-DISCOG, University of Padua, Padua, Italy; 4 Department of Medicine, Surgery and Neurosciences, Unit of General Surgery and Surgical Oncology, University of Siena, Siena, Italy

**Keywords:** Hemorrhoids, Goligher, Classification, Interobserver variability

## Abstract

**Background:**

The most widely used classification for hemorrhoidal disease (HD) is the Goligher classification, which ranks presence and severity of prolapse in four grades. Since physicians base this gradation on medical history and physical examination, it might be prone to interobserver variability. Furthermore, the gradation impacts the treatment of choice which makes reproducibility of utmost importance. The aim of this study was to determine the interobserver variability of Goligher classification among surgeons in the Netherlands.

**Methods:**

A single-choice survey was used. The first part consisted of questions concerning baseline characteristics and the use of the Goligher classification in routine clinical practice. In the second part, to assess interobserver variability, we asked gastrointestinal surgeons and residents who routinely treat HD to review 25 photographs (with given timing as during rest or push) of patients with HD and classify the gradation using the Goligher classification. The survey was sent by email on April 19, 2021 and was available online until July 5, 2021. Interobserver variability was assessed using Fleiss’ Kappa test.

**Results:**

A total of 329 gastrointestinal surgeons, fellows and residents were sent an invitation email, of whom 95 (29%) completed the survey. Among the respondents, 87% indicated that they use the Goligher classification in clinical practice. Eighty-one percent found the classification helpful and 63% classified HD according to Goligher and followed the guidelines for treatment of HD accordingly. The interobserver variability showed an overall fair strength of agreement, with a Fleiss’ Kappa (*κ*) of 0.376 (95% CI 0.373–0.380). There was a moderate agreement for grade I and IV HD with a κ statistic of 0.466 and 0.522, respectively. For grades II and III, there was a lower (fair) strength of agreement with 0.206 and 0.378, respectively.

**Conclusions:**

The fair interobserver variability is disappointing and demonstrates the need for a more reliable, and internationally accepted, classification for HD. A new classification should enable more uniformity in treating HD and in comparing outcomes of future trials and prospective registries. The protocol for a Delphi study for a new classification system is currently being prepared and led by an international research group.

## Introduction

Hemorrhoidal disease (HD) is one of the most common proctologic disorders with a prevalence up to 39% in the general population [1]. The most widely used classification system for HD is the Goligher system [2], which ranks the presence and severity of prolapse into four grades. Physicians base this gradation on medical history and physical examination, using also subjective criteria to grade HD.

The Goligher classification is used in many guidelines and thereby impacts the choices for treatment of HD worldwide. Furthermore, when comparing outcomes of different procedures for HD in studies based on the Goligher grading, its reliability and reproducibility is of utmost importance. In daily practice, it is perceived that there might be a large interobserver variability due to this mix of subjectivity and objectivity. Therefore, concerns exist about the suitability of this grading instrument to guide treatment and research. To our knowledge, the interobserver variability has never been investigated and thus remains unknown.

An important shortcoming of the Goligher classification is that it only describes a single symptom, not taking into account the number of affected piles or accompanying symptoms, i.e. pain, itching, bleeding or soiling and their impact on quality of life. Although the classification estimates the severity of prolapse, more disease burden does not automatically lead to a higher grade. This makes it difficult to evaluate and compare treatment strategies. Selection of study population is nearly always based on the Goligher classification. However, due to the abovementioned shortcomings, studies have almost never used the change in Goligher’s grade as primary endpoint, but rely on a wide variety of different end-points such as patient-reported outcome measurements or clinical outcomes, e.g. complications or recurrence symptoms defined in many different ways. Several efforts were made to classify HD in a different manner with scores based on hemorrhoidal development and symptom-based severity [3–7]. However, none of these classifications have been successful as the Goligher still remains the most used classification system in guidelines [8–10]. It has been pointed out that the simplicity of this classification is one of the main reasons for its widespread continued use over decades.

No previous study has examined the interobserver variability between physicians on assessing HD using the Goligher classification. This study aims to determine this endpoint among gastrointestinal surgeons and residents, who treat and classify hemorrhoids most frequently, and to demonstrate the need for a more reproducible and reliable classification. This could improve evaluation of treatment options for hemorrhoids and consequently improve care.

## Materials and methods

### Study design

**Fig. 1:**
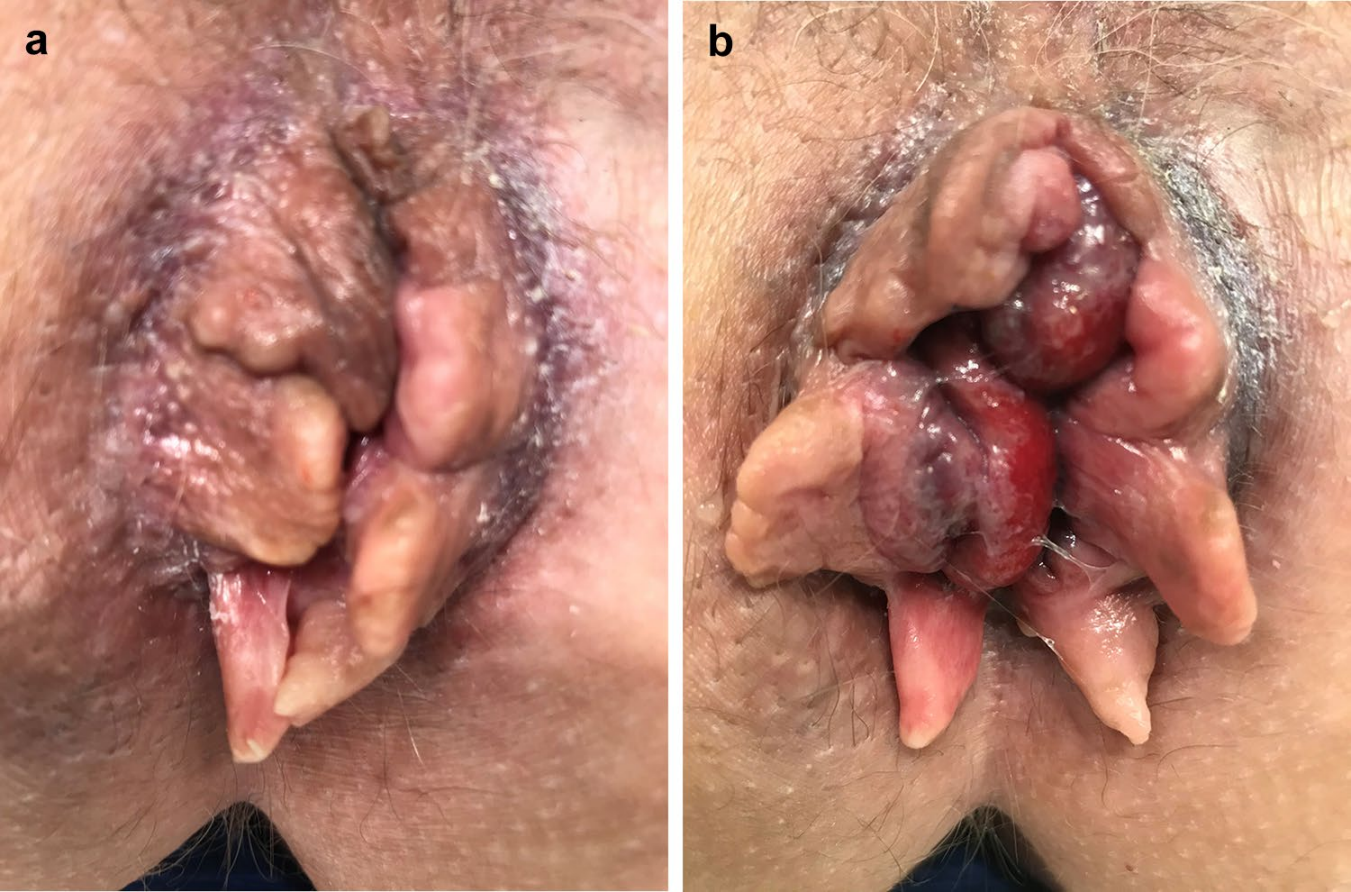
Photographs used in the survey, during rest (**a**) and strain (**b**). The patient case in the survey described a 52-year-old women with daily complaints of soiling and blood loss. She wears a panty liner. She is aware of a prolapse but that is not her main concern and she does not manually reduce it. There are no complaints of itching or pain. Results of the survey were as follows: grade I; 1 (1%), grade II; 41 (43%), grade III; 43 (45%), grade IV 10 (11%)

A single-choice survey was composed. The survey started with six questions concerning baseline characteristics and the use of the Goligher classification in routine clinical practice. Thence, the survey continued with 25 patients cases with different grades of HD. Photographs were provided with additional information concerning timing of the photo, during rest or strain, as well as medical history regarding all aspects of the Goligher classification; the presence and reducibility of prolapse. Figure 1a, b is an example of photographs used. The survey was created in Survio [11] and the definitions of the four grades were described on top of the form as a reminder (Table 1). All authors conducted a pilot for testing feasibility and validity. The finalized version was sent by email on April 19, 2021 and was available online until July 5, 2021. One email reminder was sent during the period of online availability of the survey. Observers were asked to review these cases and classify the gradation from I till IV using the Goligher classification.

**Table 1:** Definitions of the four grades of the Goligher classification

Grade	Degree of prolapse
I	No prolapse
II	Prolapse on defecation with spontaneous reduction
III	Prolapse on defecation requiring manual reduction
IV	Prolapse and irreducible

The Medical Ethics Review Committee of the Amsterdam University Medical Centers, location AMC, confirmed that the study was not subjected to the Medical Research Involving Human Subjects Act (WMO).

### Subjects

Photographs of patients with different grades of HD were obtained from electronic health records and captured during proctoscopy, before surgical intervention or taken by patients themselves. After verbal consent and a note in patients’ health record the photographs were saved anonymously. Using a two-sided alpha of 0.05 and a power of 80% a total amount of 24 photographs for 6 raters were needed to detect a statistical significant difference in kappa between 0.6 and 0.8.

### Observers

All members of the Dutch Workgroup Coloproctology, as well as Dutch gastrointestinal and colorectal surgeons, fellows and residents were invited to participate in the study. We used the email database of our previous survey concerning the management of anal fistulas among Dutch gastrointestinal surgeons [12]. Known invalid domains were removed and the list was checked globally by contact information that was retrieved from the Dutch Association of Surgery. In addition, a link to the survey was disseminated via the social media platform of the Dutch Workgroup Coloproctology as a reminder.

### Data analysis

The interobserver agreement was assessed using Fleiss’ Kappa test. Overall *k* coefficient was reported as well as the agreement for each gradation separately. Agreement was classified as follows: poor agreement (0.00–0.20), fair (0.21–0.40), moderate (0.41–0.60), substantial (0.61–0.80), and almost perfect agreement (0.81–1.00). *P* values of < 0.05 were considered significant. Data were analyzed using SPSS Statistics software version 26.0.

## Results

A total of 329 gastrointestinal surgeons, fellows and residents were sent an invitation email. Nine email addresses had an invalid domain and did not receive the invitation. Ninety-five (29%) respondents completed the survey, 86 (91%) by answering the email invitation and 9 (9%) by the web link on the social media platform of the Dutch Workgroup Coloproctology. Respondents’ characteristics and questions concerning the use of the Goligher classification in routine practice are shown in Table 2. From the respondents, 79 (84%) were gastrointestinal surgeons and 62 (65%) treated patients with HD on a weekly basis. Eighty-three (87%) used the Goligher classification when treating patients with HD and 2 respondents (2%) indicated to use both the Goligher classification and descriptive diagnosis. The majority, with 77 respondents (81%), regarded the Goligher classification a helpful tool. Of all respondents, 60 (63%) based their treatment on the Goligher classification and followed the Dutch national guideline for treatment of HD accordingly. Fifteen (16%) respondents stated that there was a broad spectrum of clinical parameters that was relevant for the choice of treatment. They indicated that the decision making was dependent on the patients’ complaints, findings at physical examination, comorbidity and patients’ preference.

**Table 2:** Respondents characteristics and questions concerning the use of the Goligher classification in routine practice

	*N* (%)
Specialty	
Surgeon	79 (84)
Fellow	7 (7)
Resident in training	8 (8)
Physician assistant/nurse practitioner	1 (1)
Regularity of treatment	
Daily	18 (19)
Weekly	62 (65)
Monthly	8 (8)
A few times in a year	7 (7)
Do you use a classification for hemorrhoids?	
Yes, the Goligher classification	83 (87)
No (e.g. descriptive diagnosis)	10 (11)
Yes, otherwise, namely.	2 (2)
The Goligher classification determines the grading on the basis of:	
Medical history	11 (12)
Physical examination	5 (5)
Proctoscopy	7 (7)
A combination of the above	72 (76)
Do you find the Goligher classification is a helpful classification	
Yes	77 (81)
No	18 (19)
To what extent do you link your treatment to the grading?	
I do not	20 (21)
I classify and I follow the guideline in the policy of treatment options	60 (63)
Otherwise, namely	15 (16)

Overall, there was only a fair strength of agreement, with a Fleiss’ Kappa (*κ*) of 0.376 (95% CI 0.373–0.380). Respondents agreed the most when it concerned grade IV HD with a κ statistic of 0.522 (moderate). Also, grade I with a *κ* of 0.466 had a moderate agreement. There was a slightly lower agreement for grade II and III HD, with a *κ* statistic of 0.206 and 0.378 (fair), respectively.

## Discussion

Although the Goligher classification appears a simple classification, as based on a single pathological parameter, the present study shows only a fair overall interobserver agreement. The classification uses the presence and severity of prolapse for grading HD, but apparently, there are unclear demarcations. The agreement for grade I and IV HD was still moderate. Differentiation between grade II and III HD appeared to be the hardest, as reflected by only fair agreement between respondents, with a κ statistic of 0.206 and 0.378, respectively.

According to the definitions in the Goligher classification, the differentiation between grade II and III HD mainly depends on the patients’ medical history (manual reduction), as prolapse and reducibility cannot always be achieved at physical examination. Patients may also mention reducibility when a concurrent anal polyp or skin tag is present and not all patients may admit to the need for manual reduction of their prolapse. This mix of morphological aspects and the subjective information may lead to different interpretation and therefore classification. Medical history and doctors’ assessment could provide well enough information in diagnosing grade I. Concerning grade IV HD, those can be misinterpreted because of the external component [13, 14]. This external component can also be a thrombosed hemorrhoid or a skin tag, which should not be classified by the Goligher system.

The initial intention of the Goligher classification is to grade HD by a single symptom that both causes complaints and defines the anatomy of the prolapse. The classification does not take into account symptoms as pain, itching, bleeding or soiling, or the actual number of prolapsing piles. This means that a single prolapsing pile can be classified the same as a full circumferential prolapse with itching, bleeding and soiling. The disparity between symptoms and grading is described by Gerjy [14]. The authors showed that HD is a polysymptomatic disease and so symptoms do not reliably relate to the Goligher classification. This hampers the adequacy of its use in research, e.g. for determining the inclusion of patients in studies evaluating different treatment strategies for HD.

Although 81% of the respondents found the Goligher classification helpful, only 63% routinely used it in their decision making process. The choice of treatment should, therefore, depend on a greater number of factors, i.e. the severity of complaints, sex, age, comorbidities and the presence of skin tags or fecal incontinence. Nevertheless, according to the current practice, a non-surgical, less invasive treatment (e.g. rubber band ligation) is the preferred choice for grade II, while surgical treatment is reserved for grade IV HD. The treatment of choice for grade III HD is still under debate and currently investigated by the Holland trial, a Dutch initiative comparing rubber band ligation and hemorrhoidectomy from a patient’s perspective [15].

Several authors have developed alternative scoring systems to overcome the abovementioned limitations [4–7, 14, 16–19]. Nevertheless, none of these well-designed classifications have been frequently, or internationally, used in clinical practice. An explanation for the difficult implementation of other classifications may rely on their relative complexity, compared to the Goligher system. Replacing this classification would be quite challenging. As suggested by Rubbini et al., since and advanced level of experience and clinical skills are commonly present in a large number of practitioners, it is recommended to initiate an innovation of such importance only if shared from the beginning by the majority of proctologists[20]. A recent survey among members of the European Society of Coloproctology (ESCP) has shown the need for a new classification system. Currently, the protocol for the development of a new classification system for HD has been initiated by our research group. The intention is to merge objective and subjective findings by performing a Delphi study that will involve clinicians and patients from ESCP member countries. Van Tol et al. already described a core outcome set for HD and showed five symptom domains that should be taken into account when studying patients with this condition [21].

The present study has a number of limitations. The response rate was low (29%). The questionnaire was sent to all members of the Dutch Coloproctology Working group that consists of members that have large experience and affiliation in treating anorectal disease, with 38 (40%) respondents coming from this workgroup. Other respondents were gastrointestinal surgeons and residents with unknown familiarity with anorectal disease. This may have influenced the outcome of the present study, but in a subgroup analysis, no differences were found.

In the design of the study, we aimed to grade HD by presenting the patients’ medical history, including the reducibility of the prolapse, and simulate the anorectal assessment by using photographs during rest and/or strain. However, the actual physical examination with digital rectal examination and, if necessary, proctoscopy is standard practice when grading by the Goligher classification. Performing digital rectal examination can provide more information about the tissue of the external component and, therefore, might distinguish between different diagnoses. Although all respondents were subjected to the same experimental conditions, this limitation in assessment may have augmented the variability of responses between participants. On the other hand, reviewing the gradation still is partially subjective and there is no right or wrong.

## Conclusions

The only fair interobserver variability in grading HD according to the Goligher classification is in accordance with the inadequacy perceived in daily practice and demonstrates the need for a more reliable, and internationally accepted grading system incorporating objective and subjective factors of HD. New classification systems should enable more uniformity of treatment of HD and a more uniform and consistent comparison of outcomes in future trials and prospective registries. The protocol for a Delphi study for a new classification system, preceded by a survey among gastrointestinal surgeons, is currently being prepared and led by an international research group.

## Declarations

### Permission to reproduce material from other sources

Yes.
